# Impact of menstrual cycle phase and oral contraceptives on sleep and overnight memory consolidation

**DOI:** 10.1111/jsr.13239

**Published:** 2020-12-21

**Authors:** Christina Paula Plamberger, Helen Elisabeth Van Wijk, Hubert Kerschbaum, Belinda Angela Pletzer, Georg Gruber, Karin Oberascher, Martin Dresler, Michael Andreas Hahn, Kerstin Hoedlmoser

**Affiliations:** ^1^ Department of Psychology Centre for Cognitive Neuroscience University of Salzburg Salzburg Austria; ^2^ Radboud University Nijmegen The Netherlands; ^3^ Department of Cell Biology Centre for Cognitive Neuroscience University of Salzburg Salzburg Austria; ^4^ Department of Psychiatry and Psychotherapy Medical University of Vienna Vienna Austria

**Keywords:** declarative memory, oral contraceptives, progesterone, sleep spindle density

## Abstract

Sleep spindles benefit declarative memory consolidation and are considered to be a biological marker for general cognitive abilities. However, the impact of sexual hormones and hormonal oral contraceptives (OCs) on these relationships are less clear. Thus, we here investigated the influence of endogenous progesterone levels of naturally cycling women and women using OCs on nocturnal sleep and overnight memory consolidation. Nineteen healthy women using OCs (M_Age_ = 21.4, *SD* = 2.1 years) were compared to 43 healthy women with a natural menstrual cycle (follicular phase: *n* = 16, M_Age_ = 21.4, *SD* = 3.1 years; luteal phase: *n* = 27, M_Age_ = 22.5, *SD* = 3.6 years). Sleep spindle density and salivary progesterone were measured during an adaptation and an experimental night. A word pair association task preceding the experimental night followed by two recalls (pre‐sleep and post‐sleep) was performed to test declarative memory performance. We found that memory performance improved overnight in all women. Interestingly, women using OCs (characterized by a low endogenous progesterone level but with very potent synthetic progestins) and naturally cycling women during the luteal phase (characterized by a high endogenous progesterone level) had a higher fast sleep spindle density compared to naturally cycling women during the follicular phase (characterized by a low endogenous progesterone level). Furthermore, we observed a positive correlation between endogenous progesterone level and fast spindle density in women during the luteal phase. Results suggest that the use of OCs and the menstrual cycle phase affects sleep spindles and therefore should be considered in further studies investigating sleep spindles and cognitive performance.

## 1 INTRODUCTION

The positive effect of sleep on memory consolidation has been elaborated intensively within the last decade (Chambers, 2017; Klinzing et al., 2019; Rasch & Born, 2013). Sleep promotes declarative memory consolidation. In particular, sleep spindles (11–16 Hz) during non‐rapid‐eye‐movement stage 2 (NREM2) seem to be an electrophysiological marker for sleep‐dependent memory consolidation (Fogel & Smith, 2011; Schabus et al., 2004). Furthermore, research over the past years suggests an effect of sex hormones on memory performance (Henderson, 2018; Le et al., 2020; Sherwin, 2012; Toffoletto et al., 2014). Progesterone levels vary across the female menstrual cycle, with low levels of progesterone during the follicular phase (starts on day 1 of menses until day 14, around ovulation) and a peak in progesterone level during the mid‐luteal cycle phase (at day 21 in a 28‐day cycle) (Le et al., 2020). Some studies observed improved memory abilities during the high progesterone luteal phase of the menstrual cycle (Ertman et al., 2011; Maki et al., 2002). Furthermore, oral contraceptive (OC) (containing very potent synthetic progestins) treatment has been associated with improved memory performance (Gogos, 2013; Gogos et al., 2014; Mordecai et al., 2008; Pletzer & Kerschbaum, 2014; Pletzer, et al., 2014a). Accordingly, the question arises of whether beneficial effects of endogenous or synthetic sex hormones on memory are in part mediated via effects of progesterone on sleep.

There is evidence that the menstrual cycle phase and the use of OCs affect sleep architecture (Baker & Driver, 2007; Baker et al., 2019). Although most studies show that sleep‐onset latency (SOL), sleep efficiency (EFF) and wakefulness after sleep onset (WASO) remain constant across the menstrual cycle, NREM2 sleep was found to be higher during the luteal phase compared to the follicular phase (Baker & Driver, 2007; Boivin & Shechter, 2010; De Zambotti et al., 2015; Driver et al., 1996, 2008). Further studies found that women using OCs have more NREM2 sleep in the active phase (OCs containing exogenous hormones for 21 days) compared to the inactive phase (placebo for 7 days) and compared to women with a natural menstrual cycle in the follicular and luteal phases (Baker et al., 2001; Driver et al., 2008). Additionally, studies revealed a minor decrease in REM sleep during the luteal phase (Baker & Driver, 2007) and an increase in total REM time in OC users compared to naturally cycling women (Burdick et al., 2002).

The strongest and most consistent effects of the menstrual cycle phase on sleep indicate an increased EEG activity in the sigma (spindle) frequency range (∼12–15 Hz), which is likely to reflect an increase in sleep spindles (Baker et al., 2007, 2012; Dijk, 1995; Driver et al., 1996), higher sleep spindle activity (defined as the mean spindle amplitude × mean spindle duration; Genzel et al., 2012), greater spindle density and longer spindles (De Zambotti et al., 2015) in naturally cycling women during the luteal phase compared to the follicular phase. Previous literature suggests that the increase of progesterone during the luteal phase extends opening times of GABA(A) (gamma‐aminobutyric acid) receptors and thereby strengthens sleep spindle activity (Fernandez & Lüthi, 2020).

So far only a few studies have investigated the relationship between sleep spindle characteristics, menstrual cycle phase and sleep‐dependent memory consolidation (Baker et al., 2019; Genzel et al., 2012; Sattari et al., 2017). Genzel and colleagues (2012) found that women had better memory consolidation and higher sleep spindle activity during diurnal sleep when they were in their luteal phase, compared to their follicular phase. These results raise the possibility that overnight memory consolidation may differ not only across the menstrual cycle but also between naturally cycling women and women using OCs. However, it remains unclear whether the use of OCs affects sleep spindle parameters and therefore sleep‐dependent memory consolidation.

Although the endogenous progesterone level in women using OCs is low, the OCs contain very potent synthetic progestins that also bind to progesterone receptors (Harada & Taniguchi, 2010; Kloosterboer et al., 1988). Synthetic progestins can have higher binding affinities to the progesterone receptors (Cabeza et al., 2015; Lovett et al., 2017) and longer elimination half‐lives than endogenous progesterone (Devoto et al., 2005; Kook et al., 2002; Kuhl, 2005; Regidor & Schindler, 2017; Schindler, 2011). The hormonal progestin exposure in some OCs is four times higher than the endogenous progesterone exposure (Lovett et al., 2017). Therefore, if sleep spindle activity is mediated by progesterone, then we expect women using OCs to show higher or equal spindle activity and sleep‐dependent memory consolidation compared with women in the luteal phase.

Based on the aforementioned literature, our study aimed at investigating sleep‐dependent declarative memory consolidation and sleep spindle density during nocturnal NREM2 sleep in three different groups of women: (1) naturally cycling women in the luteal phase (characterized by a high endogenous progesterone level [LUT]), (2) naturally cycling women in the follicular phase (characterized by a low endogenous progesterone level [FOL]), and (3) women using OCs (characterized by a low endogenous progesterone level but with potent synthetic progestins [OC]). We hypothesized that naturally cycling women during the luteal phase of their menstrual cycle and women using OCs would show an increased fast spindle density during NREM2 sleep.

Furthermore, as fast sleep spindle density is known to be related to sleep‐dependent memory consolidation, women in the luteal phase, as well as women using OCs, were expected to show better memory consolidation.

## 2 METHODS

### 2.1 Subjects

Female subjects who met the following inclusion criteria were recruited for participation: (a) women with a regular menstruation cycle, reporting a cycle duration between 21 and 35 days and a maximum variation of 7 days between the cycles (Fehring et al., 2006); (b) women using combined OCs containing dienogest and ethinylestradiol or levonorgestrel and ethinylestradiol, to get a more homogenous sample of OC users and reduce the variance of synthetic progestin exposure (Lovett et al., 2017). After the exclusion of participants with incomplete data (missing progesterone levels; missing spindle data) or progesterone as well as spindle density outliers (2 standard deviations [*SD*s] from the mean), a total of 62 participants were included in the analyses. Forty‐three of these women had a natural menstrual cycle (*n* = 16 in the follicular phase, M_Age_ = 21.4, *standard deviation [SD]* = 3.1 years, M_Cycle duration_ = 28.9, *SD* = 2.6 days; and *n* = 27 in the luteal phase, M_Age_ = 22.5, *SD* = 3.6 years, M_Cycle duration_ = 28.7, *SD* = 2.4 days) and 19 women (M_Age_ = 21.4, *SD* = 2.1 years) used OCs. Similarly to other studies (Ertman et al., 2011; Kerschbaum et al., 2017), we used a calendar‐based method to estimate the menstrual cycle phase. Based on the self‐reported onset of the last menses and the calculated cycle duration, cycle phase at the time of testing was estimated as follows. Ovulation was estimated 14 days before the expected onset of the next menses. If participants were tested before the estimated date of ovulation, they were allocated to the follicular group. If participants were tested after the estimated date of ovulation, they were allocated to the luteal group. Please note that these represent lenient criteria, because the estimated cycle phase was not confirmed by the onset of next menstruation. However, the estimated cycle phase was in concordance with sex hormone measurements. All subjects were non‐smokers, right‐handed and maintained a stable sleep–wake cycle (11:00 PM to 7:00 AM), which was controlled by daily sleep diaries and actigraphy. For participation in the study, the following exclusion criteria were defined: (a) travelling over more than three time zones within the last 3 months; (b) drug abuse in the past or present (i.e., psychoactive medicines, alcohol and/or other drugs); (c) intake of medical drugs that potentially distort or influence perception; (d) coffee consumption > 3 units per day; and (5) suffering from neurological or endocrine disorders. The study was performed in accordance with the Declaration of Helsinki and approved by the local ethics committee. Subjects gave their written informed consent before study inclusion.

### 2.2 Experimental design

**FIGURE 1:**
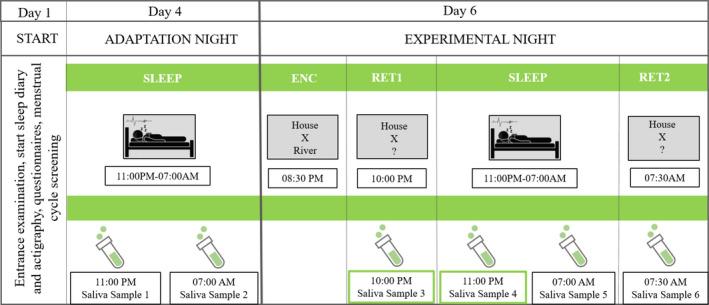
Timetable of the study. Day 1: entrance examination. Participants filled in questionnaires, underwent menstrual cycle screening and provided informed consent. Actigraphy and sleep diary were commenced and continued until day 7. Day 4: adaptation night. Overnight sleep in the laboratory, with polysomnography (PSG) and salivary samples taken pre‐ and post‐sleep. Day 6: Experimental night. Overnight sleep in the laboratory with polysomnography, word pair association tasks pre‐sleep (ENC = encoding), retrieval pre‐sleep (RET1) and salivary samples taken pre‐retrieval and pre‐sleep Retrieval post‐sleep (RET2) and salivary sample post‐sleep and post‐retrieval test were obtained. For statistical analyses, the progesterone values derived from the two samples collected before bedtime during the experimental night (saliva sample 3 and 4; framed in green) were taken

The experimental design is schematically depicted in Figure 1. All women were tested over a period of 7 days, starting with the entrance examination on day 1, where they signed the informed consent and filled in the following questionnaires: clinical evaluations of sleep quality (Pittsburgh Sleep Quality Index; Buysse et al., 1989), chronotype (D‐MEQ; Griefahn et al., 2001), personality (FPI‐R Freiburger Personality Inventory; Fahrenberg et al., 1985) and anxiety and depression (Beck Depressions‐ Inventory II and Beck Anxiety‐Inventory; Steer & Beck, 1993; Hautzinger et al., 2006); and tests of intelligence (IQ) (Advanced Progressive Matrices; Kratzmeier & Horn, 1980; Raven et al., 1998) and learning ability (VLMT; “Verbal Learning and Memory Task”; Helmstaedter et al., 1999). The VLMT consists of a standard word list with 15 semantically unrelated words (nouns), which was orally presented five times. After each presentation (R1–R5) subjects had to recall as many of the presented words as possible. The total verbal learning performance was calculated as the sum of correct words recalled over all five presentations (∑R1–R5). The free recall performance after a 30‐min time delay (R7) was calculated as the number of correct words recalled from the learning list without repeated presentation. Three days before the adaptation night and during the whole study (until day 7) the sleep–wake rhythm was controlled by actigraphy (Cambridge Neurotechnology Actiwatch©, Cambridge, UK) and a daily sleep diary (adapted from Saletu et al., 1987). To check for menstrual cycle phase women had to complete a menstrual cycle screening, where they self‐reported the date of their last three menstruations and usual cycle duration (the menstrual cycle screening was adapted from Pletzer, et al., 2014a). On day 4 (adaptation night) and day 6 (experimental night), polysomnography (PSG) was recorded during sleep between ± 11:00 PM and ± 7:00 AM (±8 h of sleep). In addition, six hormone saliva samples (1, pre‐sleep adaptation night; 2, post‐sleep adaptation night; 3 and 4, pre‐sleep experimental night; 5 and 6, post‐sleep experimental night) (cf. Figure 1 for details) were collected to investigate progesterone levels.

On day 6 (experimental night, around 8:30 PM), the participants performed a declarative learning task in two encoding blocks, each of 27 min (see Schabus et al., 2004 for details). After the two encoding blocks, the participant took a 10‐min break, which was followed by the first retrieval block around 10:00 PM (RET1). Only the first word of the word pairs learned during the encoding block was shown and the participant had to remember the corresponding second word. They did not receive any feedback during the retrieval blocks. Whenever the participant remembered the corresponding word, she was asked to press the space bar and report the corresponding word. The next morning on day 7, at around 7:30 AM, the women performed the second retrieval task (RET2).

Declarative memory performance was calculated according to Schabus et al. (2004). The recall score consisted of (a) number of correctly recalled word pairs and (b) semantic (unambiguous) correct word pairs (e.g. ‘‘boot’’ or ‘‘sandal’’ instead of ‘‘shoe’’). Correct word pairs were weighted by 1 and semantically correct pairs were weighted by 0.5. Recall performance (RET1 = retrieval block in the evening or RET2 = second retrieval block in the morning) was expressed as percentage (recall score/total count of word pairs * 100). Total count of word pairs was 160. Overnight memory consolidation was calculated by subtracting pre‐sleep recall performance (RET1) from post‐sleep recall performance (RET2).

### 2.3 Polysomnography

Polysomnography was recorded using a 32‐channel Neuroscan system (NeuroScan, El Paso, Texas, USA) and data were analysed using the Brain Vision Analyzer 2 Software (Version 2.04; Brain Products GmbH, Gilching, Germany). Recordings were derived from 10 scalp EEG channels (F3, Fz, F4, C3, C4, P3, Pz, P4, O1 and O2), left and right horizontal electrooculogram (EOG) channels, two vertical EOG channels and two chin electromyogram (EMG) channels. Data were recorded against the online reference Cz and re‐referenced against the contralateral mastoids A1 and A2. Impedance was kept below 10 kΩ. A low‐pass filter of 100 Hz, a 0.05‐Hz high‐pass filter and a notch filter of 50 Hz were applied to the data. The PSG signal was recorded with a sampling rate of 500 Hz. Sleep stages were scored automatically (Somnolyzer 24 × 7, Koninklijke Philips N.V., Eindhoven, the Netherlands) and visually cross‐validated by an expert sleep scorer according to criteria of the American Academy of Sleep Medicine (Iber et al., 2007). Sleep spindles during NREM2 sleep at F3, F4, C3 and C4 were analysed using the software ASK Analyzer (The Siesta Group, Vienna, Austria), whereby the twofold spindle detection was based on an automatic algorithm. First, “possible” spindle events were detected by the low‐specificity but high‐sensitivity band‐pass method (Schimicek et al., 1994). The following criteria were used for this step: (a) 11–16‐Hz band‐pass filtering, (b) amplitude > 12 µV, (c) duration 0.3–2 s and (d) controlling for muscle (30–40 Hz) and alpha (8–12 Hz) artefacts. Second, the detected “possible” spindle events were further evaluated in order to increase specificity. From all “possible” spindle episodes, “certain” spindle episodes were identified by means of a linear discriminate analysis (LDA) trained on visually scored spindles. The LDA uses the five log‐transformed characteristics (spindle duration and mean amplitudes in four frequency bands: spindle, theta, alpha and fast beta) of the “possible” spindle episodes. Only “certain” spindle events with a discriminant score > 1.7, which corresponds to a specificity of 98%, were used for analyses. A specificity of 98% is similar to visual scorers (for more details, see Anderer et al., 2005). Furthermore, the spindles identified by the algorithm were visually inspected to ensure valid detections. A distinction was made between slow spindles (11–13 Hz) and fast spindles (13–15 Hz). Spindle density was calculated as the mean number of spindles per minute of NREM2 sleep (N/min). Spindle density was calculated for each electrode separately and afterwards averaged for frontal (F3,F4) and central (C3,C4) derivations.

### 2.4 Hormonal samples

Overall, six saliva samples were collected: before and after the adaptation night (pre‐ and post‐sleep), two before sleep during the experimental night (day 6), and two after sleep on day 7 (cf. Figure 1). Progesterone level (pg/ml) was quantified using the Enzyme‐linked Immunosorbent Assay Kit (ELISA) according to the recommendation of the provider (Demeditec Diagnostics GmBH, Germany). All salivary samples were stored in sterile centrifuge tubes at −20 degree Celsius until analysis. Particles in saliva samples were removed by centrifugation before progesterone quantification. For statistical analyses the progesterone values derived from the two samples taken before bedtime during the experimental night (10:00 PM; 11:00 PM) were averaged to achieve a more robust estimate of endogenous progesterone level (Demeditec Diagnostics GmbH, user´s manual, 2018; Pletzer, et al., 2014b).

### 2.5 Statistical analyses

Statistical analyses were performed using SPSS 24 (SPSS Inc., Chicago, Illinois, USA). To test whether the data were normally distributed, Shapiro‐Wilk tests were used. Note that we had to exclude six participants because their spindle density values exceeded the group mean by more than two SDs and 14 participants because one or more of the progesterone values exceeded the group mean by more than two SDs. Analyses were mainly based on one‐way and repeated measure analyses of variance (ANOVA) or Kruskal‐Wallis tests, depending on the distribution of the data. The significance level was set to *p* < .05; *p*‐values ≤ 0.10 were reported as statistical trends. Partial eta squared (η_p_^2^) (for parametric tests) and the correlation coefficient (*r*) (for non‐parametric tests) are reported for effect sizes. Greenhouse‐Geisser correction was used in case the assumption of sphericity was violated. For post‐hoc comparisons of mean values, parametric tests (*t* tests) or Mann–Whitney *U*‐tests were applied, depending on the distribution of the data. Post‐hoc tests are Bonferroni corrected. Post‐hoc tests that did not survive Bonferroni correction are marked by a ‘†’. For correlations, either the Pearson coefficient (*r*_P_) for all normally distributed variables or Spearman rho coefficients (*r*_S_) for all not normally distributed variables are reported.

## 3 RESULTS

### 3.1 Progesterone level

**FIGURE 2:**
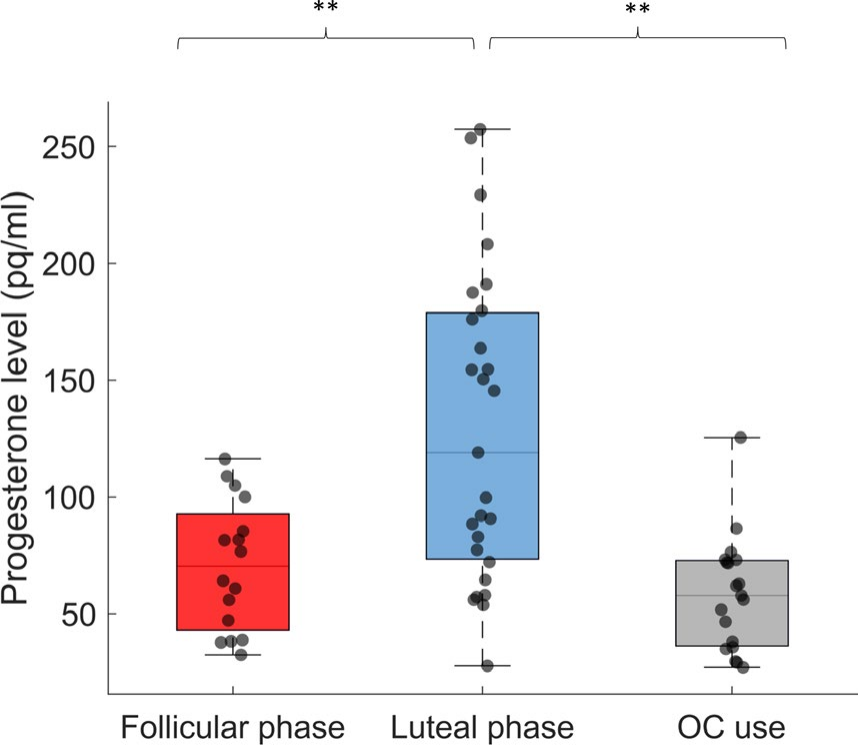
Pre‐sleep progesterone levels (pg/ml) during the experimental night for naturally cycling women in the follicular phase (*n* = 16, red, M = 70.6, *SD*= 27.8) and in the luteal phase (*n* = 27, blue, M = 129.2, *SD* = 65.8) as well as for women using OCs (*n* = 19, grey, M = 58.4, *SD* = 24.5). Box‐and‐whisker plot illustrates the population data together with their median and 25/75% percentiles, including individual data points. **p < .01

A one‐way ANOVA revealed that the progesterone level differed significantly between the three groups of women (*F*(2,59) = 14.472, *p* < .001, $\eta_{p}^{2}$ = 0.329). As depicted in Figure 2, post‐hoc independent sample *t* tests showed a significantly higher progesterone level in naturally cycling women during the luteal phase compared to naturally cycling women in the follicular phase (*t*(41) = −4.058, *p* < .001, $\eta_{p}^{2}$ = 0.287) and compared to women using OCs (*t*(44) = −5.114, *p* < .001, $\eta_{p}^{2}$ = 0.373). Progesterone levels did not differ significantly between naturally cycling women in the follicular phase and women using OCs (*t*(33) = 1.385, *p* = .175, $\eta_{p}^{2}$ = 0.055).

### 3.2 Declarative memory performance

**FIGURE 3:**
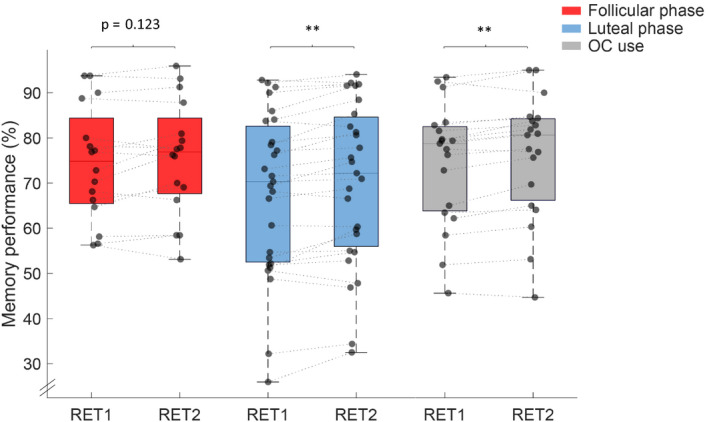
Pre‐sleep (RET1) and post‐sleep (RET2) memory performance (%) for women with a natural menstrual cycle in the follicular phase (*n* = 16, red) and luteal phase (*n* = 27, blue) and women using OCs (*n* = 19, grey). Box‐and‐whisker plot illustrates the population data together with their median and 25/75% percentiles, including individual data points. **p < .01

Recall performance in the evening (RET1) and in the morning (RET2) was positively correlated in all three groups (FOL: *r*_p_(14) = .972, *p* < .001; LUT: *r*_p_(25) = .987, *p* < .001; OC: *r*_p_(17) = .992, *p* < .001). Further, there were no group differences for recall performance in the evening (RET1: *F*(2,59) = 1.412, *p* = .252, $\eta_{p}^{2}$ = 0.046) or in the morning RET2 (*F*(2,59) = 1.138, *p* = .327, $\eta_{p}^{2}$ = 0.037; cf. Table 1 and Figure 3). To investigate sleep‐related memory consolidation a 2 × 3 repeated measure ANOVA with the factors TIME (RET1 and RET2) and GROUP (FOL, LUT and OC) was conducted. A main effect for TIME (*F*(1,59) = 23.105, *p* < .001, $\eta_{p}^{2}$ *= *0.281) was found. Neither the between‐subjects effect GROUP (*F*(2,59) = 1.280, *p* = .286, $\eta_{p}^{2}$ *= *0.042), nor the interaction TIME × GROUP (*F*(2,59) = 0.595, *p* = .555, $\eta_{p}^{2}$ *= *0.020) was significant. Descriptive post‐hoc dependent sample *t* tests revealed a significant change in memory performance overnight in women during the luteal phase (*t*(26) = −3.770, *p* = .001, $\eta_{p}^{2}$ = 0.353) and women using OCs (*t*(18) = −4.008, *p* = .001, $\eta_{p}^{2}$ = 0.472) but not in women during the follicular phase (FOL: (*t*(15) = −1.634, *p* = .123, $\eta_{p}^{2}$ = 0.151). All calculations were controlled for effects of age, intelligence (IQ) and learning ability (VLMT), as well as progesterone levels (cf. Table 1, Supplementary Material p.1 and Table S1). Only one of these control calculations revealed a trend for a significant result, indicating that women using OCs showed a moderate positive correlation (*r*_p_(17) = .454, *p* = .051) between progesterone level and overnight memory consolidation (cf. Table S1).

**TABLE 1:** Scores of memory performance and memory consolidation as well as scores of intelligence (IQ) and learning ability (VLMT)

	FOL (*n* = 16)	LUT (*n* = 27)	OC (*n* = 19)	FOL versus LUT			FOL versus OC			OC versus LUT		
				*t*	*p*	$\eta_{p}^{2}$	*t*	*p*	$\eta_{p}^{2}$	*t*	*p*	$\eta_{p}^{2}$
Behavioural data												
RET1	74.47 ± 12.62	67.84 ± 18.02	74.49 ± 13.41	1.294	.203	0.039	−0.004	.997	0.000	1.364	.180	0.041
RET2	75.70 ± 12.71	69.95 ± 17.94	76.10 ± 13.55	1.123	.268	0.030	−0.089	.929	0.000	1.261	.214	0.035
ΔRET2 − RET1	1.23 ± 3.01	2.12 ± 2.92	1.61 ± 1.75	−0.953	.346	0.022	−0.447	.659	0.006	−0.733	.468	0.012
IQ	113.84 ± 13.12	112.81 ± 16.19	117.71 ± 9.51	0.215	.831	0.001	−1.009	.320	0.030	1.287	.205	0.036
∑R1−5	65.00 ± 4.90	63.56 ± 7.33	61.89 ± 5.72	0.699	.488	0.012	1.706	**.097**	0.081	−0.825	.414	0.015
				***Z***	***p***	***r***	***Z***	***p***	***r***	***Z***	***p***	***r***
R7	14.44 ± 0.96	13.85 ± 1.49	14.00 ± 1.29	−1.555	.120	−.237	−1.051	.293	−.178	−0.415	.678	−.061

Note:

Values are expressed as mean ± *SD*. Statistical trends are marked in bold.

Abbreviations: FOL, follicular phase; LUT, luteal phase; OC, oral contraceptive use; VLMT, Verbal Learning and Memory Task.

Word pair association task: RET1 = pre‐sleep recall performance, RET2 = post‐sleep recall performance, ΔRET2‐RET1 = memory consolidation. VLMT: ∑R1‐5 = total learning performance, R7 = free recall performance after a 30‐min time delay.

### 3.3 Sleep architecture

For each sleep architecture parameter (time in bed [TIB], total sleep time [TST], sleep‐onset latency [SOL], sleep efficiency [EFF], wake after sleep onset [WASO], percentage of sleep stage NREM1, NREM2 and NREM3, and percentage of rapid eye movement sleep [REM%]), we calculated a one‐way ANOVA or Kruskal‐Wallis test comparing the three groups of women. Results are depicted in Table 2 and showed a significant group effect for the percentage of REM (*F*(2,59) = 4.399, *p* = .017, $\eta_{p}^{2}$ *= *0.130) and NREM1 sleep (H(2)=6.292, *p* = .043). Post‐hoc independent sample *t* tests revealed that women using OCs showed less REM sleep (%) compared to naturally cycling women during the luteal phase (*t*(44) = −3.396, *p* = .001, $\eta_{p}^{2}$ = 0.208) and by trend compared to naturally cycling women during the follicular phase (*t*(33) = 1.869, *p* = .074, $\eta_{p}^{2}$ = 0.096). Post‐hoc Mann–Whitney *U*‐tests (Bonferroni corrected: 0.05/3 = 0.017) revealed that women using OCs showed by trend less NREM1 sleep compared to naturally cycling women during the luteal (*Z* = −1.707, *p* = .088, *r* = .252) or follicular phase (*Z* = −2.252, *p* = .024, *r* = .381).

**TABLE 2:** Sleep architecture

	FOL (*n* = 16)	LUT (*n* = 27)	OC (*n* = 19)	FOL versus LUT			FOL versus OC			OC versus LUT		
				*Z*	*p*	*r*	*Z*	*p*	*r*	*Z*	*p*	*r*
Sleep architecture												
TIB (min)	467.22 ± 55.53	480.00 ± 3.22	466.34 ± 63.66	−0.580	.562	−.088	−0.912	.362	−.154	−0.366	.714	−.054
TST (min)	445.47 ± 55.94	458.11 ± 16.78	431.79 ± 70.68	−0.251	.802	−.038	−0.480	.631	−.081	−0.703	.482	−.104
SOL (min)	19.50 ± 7.21	18.30 ± 16.99	16.76 ± 10.14	−1.521	.128	−.232	−0.895	.371	−.151	−0.201	.841	−.030
EFF (%)	95.29 ± 2.78	95.44 ± 3.52	92.65 ± 8.41	−0.440	.660	−.067	−0.364	.716	−.062	−0.770	.441	−.114
WASO (min)	12.22 ± 9.55	11.30 ± 7.72	22.95 ± 35.43	−0.126	.900	−.019	−0.365	.715	−.062	−0.301	.763	−.044
NREM1(%)	12.52 ± 4.49	10.62 ± 3.68	9.59 ± 6.35	−1.357	.175	−.207	−2.252	**.024^†^**	−.381	−1.707	**.088**	−.252

				*t*	*p*	$\eta_{p}^{2}$	*t*	*p*	$\eta_{p}^{2}$	*t*	*p*	$\eta_{p}^{2}$
NREM2 (%)	38.51 ± 8.35	40.80 ± 7.21	42.13 ± 6.76	−0.950	.347	0.022	−1.418	.165	0.057	0.632	.530	0.009
NREM3 (%)	32.39 ± 11.87	31.34 ± 9.05	35.77 ± 10.82	0.327	.745	0.003	−0.880	.385	0.023	1.507	.139	0.049
REM (%)	16.58 ± 7.55	17.23 ± 4.57	12.50 ± 4.77	0.312	.758	0.002	1.869	**.074**	0.096	−3.396	**.001** **	0.208

Note:

Values are expressed as mean ± standard deviation [*SD*].

*p*‐values that did not survive Bonferroni correction (for fast sleep spindle density: 0.05/3 = 0.017) are marked by a ‘^†^’. Significant results and statistical trends are marked in bold.

Abbreviations: EFF (%), sleep efficiency; FOL, follicular phase; LUT, luteal phase; NREM1 (%), percentage of non‐rapid eye movement (NREM) sleep stage 1; NREM2 (%), percentage of sleep stage NREM2; NREM3 (%), percentage of sleep stage NREM3; OC, oral contraceptive use; REM (%), percentage of rapid eye movement (REM) sleep; SOL (min), sleep‐onset latency to N2; TIB (min), time in bed; TST (min), total sleep time; WASO (min), wake after sleep onset.

** *p*<.01.

### 3.4 Sleep spindle density

**FIGURE 4:**
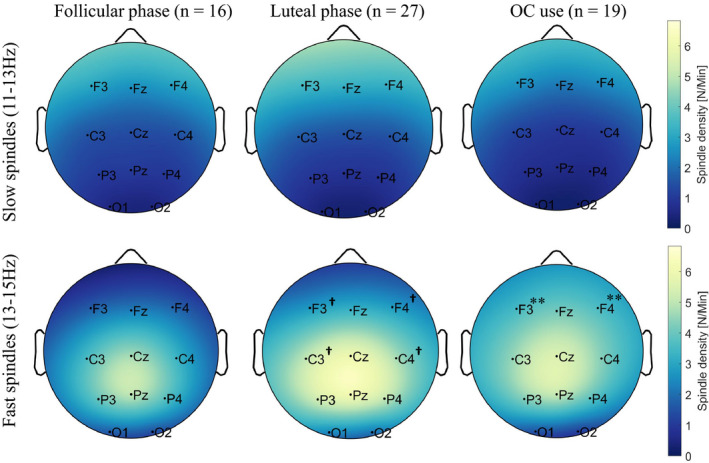
Sleep spindle density topography during the experimental night (N/min; colour scaled) for naturally cycling women in their follicular phase (*n* = 16) and luteal phase (*n* = 27) as well as women using OCs (*n* = 19). Note that bright colours indicate higher sleep spindle density. Fast spindle density (lower row) was higher at central compared to frontal electrodes, whereas slow spindle density (upper row) was higher at frontal compared to central electrode sites. Fast spindle density was higher in OC users compared to women during the follicular phase and by trend in women during the luteal phase compared to the follicular phase. ** Represents significant differences (*p* < .01) in sleep spindle density in women using OCs compared to women during the follicular phase of the menstrual cycle (OC > follicular). **^†^**Represents by trend a difference (*p* < .10) in sleep spindle density in women during the luteal phase compared to women during the follicular phase of the menstrual cycle (luteal > follicular)

A repeated measures ANOVA with the within‐subject factor LOCATION (frontal or central) and the between‐subjects factor GROUP (FOL, LUT or OC) was calculated separately for fast and slow spindle density. Both ANOVAs revealed a significant main effect for LOCATION (slow spindle density: *F*(1,59) = 213.083, *p* < .001, $\eta_{p}^{2}$ *= *0.783; fast spindle density: *F*(1,59) = 137.316, *p* < .001, $\eta_{p}^{2}$ *= *0.699), indicating higher fast spindle density at central electrode sites and higher slow spindle density at frontal electrode sites (cf. Figure 4 and Table 3). The main between‐subjects effect GROUP only reached significance for fast spindle density (*F*(2,59) = 3.639, *p* = .032, $\eta_{p}^{2}$ *= *0.110) and not for slow spindle density (*F*(2,59) = 1.130, *p* = .330, $\eta_{p}^{2}$ *= *0.037). Post‐hoc independent sample *t* tests (Bonferroni corrected: 0.05/3 = 0.017) showed significant higher fast spindle density in naturally cycling women during the luteal phase compared to women during the follicular phase (*t*(41) = −2.732, *p* = .009, $\eta_{p}^{2}$ *= *0.154), as well as by trend in OC users compared to naturally cycling women during the follicular phase (*t*(33) = −2.479, *p* = .019^†^, $\eta_{p}^{2}$ *= *0.157). Furthermore, a significant interaction between LOCATION × GROUP was found for fast spindle density (*F*(2,59) = 8.334, *p* = .001, $\eta_{p}^{2}$ *= *0.220) but not for slow spindle density (*F*(2,59) = 1.260, *p* = .291, $\eta_{p}^{2}$ *= *0.041). Post‐hoc independent sample *t* tests (Bonferroni corrected: 0.05/6 = 0.008) indicated that women using OCs showed significantly higher frontal fast spindle density compared to naturally cycling women during the follicular phase (*t*(33) = −3.352, *p* = .002, $\eta_{p}^{2}$ *= *0.254). Furthermore, naturally cycling women during the luteal phase showed by trend higher frontal (*t*(41) = −2.473, *p* = .018^†^, $\eta_{p}^{2}$ *= *0.130) and central (*t*(41) = −2.643, *p* = .012^†^, $\eta_{p}^{2}$ *= *0.146) fast spindle density compared to naturally cycling women during the follicular phase. No differences in fast spindle density were found when comparing naturally cycling women during the luteal phase with women using OCs (*t*(44) = 0.285, *p* = .777, $\eta_{p}^{2}$ *= *0.002; cf. Figure 4 and Table 3). To analyse whether these menstrual cycle‐dependent differences in fast sleep spindle density are related to endogenous progesterone levels, we additionally calculated correlations between progesterone level and spindle density. Naturally cycling women during the luteal phase showed a trend for a positive association between frontal fast spindle density and progesterone level (*r*_p_(25) = .346, *p* = .077). For follicular phase women and OC users, we did not detect a significant correlation (FOL: *p* > .7; OCs: *p* > .4; for details cf. Table 4). Regarding our last hypothesis that fast sleep spindle density is related to sleep‐dependent memory consolidation, we calculated correlations between fast sleep spindle density and overnight memory change for the three groups (FOL: all *p* > .3; LUT: all *p* > .1; OC: all *p* > .5; cf. Table S2). All calculations were controlled for effects of intelligence (IQ) and learning ability (VLMT) (cf. Table S2).

**TABLE 3:** Spindle density from fast and slow sleep spindles at frontal and central electrode positions for the three tested groups (follicular phase, luteal phase and oral contraceptive users)

	FOL (*n* = 16)	LUT (*n* = 27)	OC (*n* = 19)		FOL versus LUT			FOL versus OC			OC versus LUT		
					*t*	*p*	$\eta_{p}^{2}$	*t*	*p*	$\eta_{p}^{2}$	*t*	*p*	$\eta_{p}^{2}$
Spindle density													
Slow frontal	2.95 ± 1.49	3.35 ± 1.79	2.57 ± 1.51	−0.747		.460	0.013	0.749	.459	0.017	−1.548	.129	0.052
Slow central	1.49 ± 1.21	1.59 ± 1.22	1.17 ± 0.81	−0.254		.801	0.002	0.919	.367	0.025	−1.413	.165	0.043
Fast frontal	1.77 ± 1.04	2.77 ± 1.40	3.54 ± 2.01	−2.473		**.018** ^†^	0.130	−3.352	**.002** **	0.254	1.547	.129	0.052
Fast central	3.45 ± 1.34	4.89 ± 1.92	4.42 ± 2.23	−2.643		**.012** ^†^	0.146	−1.587	.123	0.071	−0.766	.448	0.013

Note:

Values are expressed as mean ± standard deviation [*SD*].

*p*‐values that did not survive Bonferroni correction (for fast sleep spindle density: 0.05/6 = 0.008) are marked by a ‘^†^’. Significant results and statistical trends are marked in bold.

Abbreviations: central, electrodes C3, C4; Fast, sleep spindle density (N/min) for fast spindles (13–15 Hz); FOL, follicular phase; frontal, electrodes F3, F4; LUT, luteal phase; OC, oral contraceptive use; Slow, sleep spindle density (N/min) for slow spindles (11–13 Hz).

** *p* < .01.

**TABLE 4:** Correlations between progesterone level and fast sleep spindle density

Spindle density	FOL (*n* = 16)	LUT (*n* = 27)	OC (*n* = 19)
Fast frontal			
*r*	−.028	.346	−.162
*p*	.919	**.077**	.508
Fast central			
*r*	−.047	.194	−.183
*p*	.862	.332	.454

Abbreviations: central, electrodes C3, C4; Fast, sleep spindle density (N/min) for fast sleep spindle density (13–15 Hz); FOL, follicular phase; frontal, electrodes F3, F4; LUT: luteal phase; OC, oral contraceptive use. Statistical trends are marked in bold.

## 4 DISCUSSION

The aim of this study was to investigate the effects of menstrual cycle phase and the use of OCs on sleep‐dependent declarative memory consolidation and sleep spindle density during nocturnal NREM2 sleep.

### 4.1 Progesterone level

As expected from the literature (De Bondt et al., 2013; Le et al., 2020), we observed that endogenous progesterone levels are highest in naturally cycling women during the luteal phase compared to naturally cycling women during the follicular phase and women using OCs (cf. Figure 2).

### 4.2 Declarative memory performance

There is evidence showing that hormonal fluctuations across the menstrual cycle and the use of OCs modulate memory performance (Henderson, 2018; Le et al., 2020; Sherwin, 2012; Toffoletto et al., 2014). Although some studies reported better memory performance during the luteal phase of the menstrual cycle (characterized by a high endogenous progesterone level) (Ertman et al., 2011; Maki et al., 2002) and in women using OCs (characterized by a low endogenous progesterone level but with potent synthetic progestins; Gogos, 2013; Gogos et al., 2014; Mordecai et al., 2008; Pletzer & Kerschbaum, 2014; Pletzer, et al., 2014a), others did not (Mihalik et al., 2009; Protopopescu et al., 2008). Studies investigating the impact of menstrual cycle phase and the use of OCs on sleep‐dependent declarative memory consolidation are scarce (Genzel et al., 2012, 2014). In the present study, no differences were found between the three groups of women (FOL, LUT and OC) for memory performance (RET1, RET2; cf. Figure 3) or for control variables such as intelligence (IQ) or learning ability (VLMT). We found that memory performance improved overnight in all three groups. Although repeated measure ANOVAs showed no group differences in overnight memory consolidation, descriptive post‐hoc dependent sample *t* tests revealed a highly significant change in memory performance overnight in naturally cycling women during the luteal phase (*p* = .001) and women using OCs (*p* = .001) but not in naturally cycling women during the follicular phase (*p* = .123; cf. Figure 3). To evaluate why the difference between pre‐ and post‐sleep performance was not significant in women during the follicular phase and whether this could be caused by lower statistical power, a post‐hoc power analysis was calculated. Because the achieved statistical power for women during the follicular phase is as high as for the other two groups (for post‐hoc power analyses cf. Table S3), we conclude that the positive effect of sleep on memory consolidation after a declarative learning task is indeed more pronounced in women using OCs and naturally cycling women during the luteal phase, compared to naturally cycling women during the follicular phase. Similarly, Genzel and colleagues (2012) found enhanced declarative memory consolidation in naturally cycling women during the luteal phase, but not during the follicular phase, during a daytime nap compared to a wake condition. In contrast to our study, Genzel and colleagues (2012) used a within‐subject design and therefore might have revealed larger menstrual cycle effects on sleep‐dependent memory consolidation because within‐subject designs have more power because of a decrease in inter‐subject variability (Charness et al., 2012). Although we controlled for confounding variables such as age, intelligence (IQ) or learning ability (VLMT) and only included healthy women without any psychiatric or neurological disorders, it is still possible that the three groups differ in an aspect that was not matched here. For example, hormone levels of estradiol, which is also known to be positively associated with declarative memory performance (Genzel et al., 2012), or dopamine, which modulates higher cognitive functions (Hidalgo‐Lopez & Pletzer, 2017), could be a source of variance between subjects.

Based on our results and the study by Genzel and colleagues (2012), it seems that the varying sex hormones during the menstrual cycle and the use of synthetic sex hormones have an effect on declarative memory consolidation following sleep.

### 4.3 Sleep architecture

Besides the unexpected significantly higher amount of REM sleep in naturally cycling women during the luteal phase compared to women using OCs, and the minor increase in NREM1 sleep in naturally cycling women during the luteal and follicular phases compared to women using OCs, sleep architecture did not differ between the three groups. We failed to replicate earlier findings showing a minor decrease in REM sleep (Baker & Driver, 2007; Driver et al., 1996) during the luteal compared to the follicular phase or an increase in total REM time (Burdick et al., 2002) in OC users compared to naturally cycling women. Note that Burdick and colleagues (2002), who found a higher REM time in OC users compared to naturally cycling women, did not distinguish between different menstrual cycle phases and had a very small sample size of *n* = 9 women using OCs without any information about what kind of OCs (monophasic or triphasic) were used. Interestingly, Baker and colleagues (2012) reported that high progesterone levels in naturally cycling women correlated with a decreased percentage of REM sleep. If synthetic progestins (levonorgestrel and dienogest) act like endogenous ones, we would expect a decrease in REM sleep in OC users.

Other studies found an increase in NREM2 sleep in naturally cycling women tested during the luteal phase compared to the same women tested during the follicular phase (Driver et al., 1996, 2008) and in OC users compared to naturally cycling women (Baker et al., 2001; Driver et al., 2008). Although in our study NREM2 sleep did not differ significantly between the three groups, descriptive data are in line with previous findings showing higher amounts of NREM2 sleep in women during the luteal phase compared to women during the follicular phase and in OC users compared to naturally cycling women (cf. Table 2).

### 4.4 Sleep spindle density

The strongest and most consistent effects of the menstrual cycle phase on sleep spindles are indicated by (a) an increased EEG activity in a frequency range corresponding to the upper frequency range of the sleep spindles (∼13–15 Hz), which is likely to reflect an increase in fast sleep spindles (Baker et al., 2007, 2012; Dijk, 1995; Driver et al., 1996); (b) generally higher sleep spindle activity (defined as the mean spindle amplitude × mean spindle duration; Genzel et al., 2012); (c) enhanced sleep spindle density and (d) longer sleep spindle duration (De Zambotti et al., 2015) in naturally cycling women during the luteal compared to the follicular phase. In line with these findings, our data show higher frontal and central fast sleep spindle density in naturally cycling women during the luteal phase compared to women during the follicular phase (cf. Figure 4). We further found larger fast spindle density (especially at frontal electrodes) in women using OCs compared to naturally cycling women in the follicular phase. These findings suggest that menstrual cycle‐dependent oscillations in progesterone and application of synthetic sex hormones modulate fast sleep spindle density.

The mechanisms of how (endogenous and synthetic) sex hormones affect sleep spindle density are not fully understood. In previous studies the effects of the menstrual cycle on sleep spindle parameters are often explained by the effect of progesterone metabolites on GABA(A) receptors (Baker et al., 2007, 2012, 2019; Fernandez & Lüthi, 2020; Plante & Goldstein, 2013). This hypothesis is partly supported by findings from human and animal studies showing that progesterone administration and allopregnanolone, a progesterone ‐metabolite, increase NREM sleep and EEG activity in the sleep spindle frequency range (Damianisch et al., 2001; Friess et al., 1997; Lancel, 1999; Lancel et al., 1996). The progesterone‐metabolite allopregnanolone derives from endogenous progesterone and synthetic progestins and enhances the effects of GABA, an inhibitory neurotransmitter, via the modulation of GABA(A) receptors (Bernardi et al., 2006; Harrison et al., 1987; Paul et al., 2020). GABAergic thalamic reticular neurons play a crucial role in sleep spindle generation (De Gennaro & Ferrara, 2003). Because we found a larger spindle density in the luteal phase of the menstrual cycle (characterized by a high endogenous progesterone level) and by trend a positive correlation between progesterone level and frontal fast spindle density in naturally cycling women during the luteal phase (characterized by a high progesterone level), we conclude that progesterone metabolites increase sleep spindle density. Fast spindle density was even more pronounced in women using OCs. This observation is not surprising since progestins, synthetic gestagens in OCs, are metabolized more slowly (Devoto et al., 2005; Kook et al., 2002; Kuhl, 2005; Schindler, 2011) and in different ways than endogenous progesterone (Giatti et al., 2016). Progestins also bind to progesterone receptors (Harada & Taniguchi, 2010; Kloosterboer et al., 1988) and sometimes have higher binding affinities to the progesterone receptors (Cabeza et al., 2015; Harada & Taniguchi, 2010; Lovett et al., 2017; Regidor & Schindler, 2017). In addition, the synthetic progestins are consistently high over the 3 weeks of intake, whereas the progesterone levels in naturally cycling women rise after ovulation, reach a peak during the mid‐luteal phase and decrease before the next menses (Le et al., 2020). The hormonal progestin exposure in some OCs is four times higher than the endogenous progesterone exposure (Lovett et al., 2017). Consequently, the progesterone‐dependent impact on neural networks is magnified. Interestingly, in line with this, Plante and Goldstein (2013) found that female patients taking medroxyprogesterone acetate, a synthetic progesterone, showed higher sleep spindle density compared to patients without any hormone therapy.

### 4.5 Memory consolidation and spindle density

Previous literature suggests that sleep spindles benefit declarative memory consolidation (e.g., Gais et al., 2002; Schabus et al., 2004). An increase in spindle density has been shown during a night following a declarative memory task compared to a control night following a non‐learning task and spindle density was positively associated with pre‐ and post‐sleep memory performance (Gais et al., 2002). Furthermore, better declarative memory performance after sleep was only found for subjects showing enhanced spindle activity (a measurement combining spindle duration and amplitude) during the experimental night compared to a non‐learning control night (Schabus et al., 2004). There is accumulating evidence demonstrating fast sleep spindles to have a positive effect on sleep‐dependent declarative memory consolidation (Cox et al., 2014; Groch et al., 2017; Mölle et al., 2011). For example, Groch and colleagues (2017) used targeted memory reactivations and demonstrated that memory cues, related to prior knowledge, benefit successful memory consolidation during sleep and were associated with increased fast spindle activity.

We did not find significant correlations between sleep spindle density and sleep‐dependent memory consolidation. However, our design differs from the above‐mentioned studies in three aspects: (a) rather than measuring the spindle density (number of spindles per minute), Schabus and colleagues (2004) used the measure sleep spindle activity (capturing the duration as well as amplitude of identified spindles); (b) whereas Schabus and colleagues (2004) and Gais and colleagues (2002) found that the percentage increase in spindle activity or spindle density from a non‐learning control night to an experimental learning night was associated with an increase in memory performance, our design is lacking a proper control condition to investigate changes in sleep spindle activity from a non‐learning to a learning night; and (c) Groch and colleagues (2017) investigated the relationship between sleep spindles and memory consolidation by utilizing targeted memory reactivation. Note that although we did not find a direct association between spindle density and overnight memory consolidation, our data revealed that in those groups where fast spindle density was increased (in women during the luteal phase and in women using OCs) descriptive post‐hoc dependent sample *t*‐tests showed a highly significant change in memory performance overnight compared to the follicular group with significantly lower fast spindle density, along with a non‐significant increase in overnight memory performance. Therefore, we suggest that menstrual cycle‐dependent oscillations of the sex hormone progesterone and the use of synthetic sex hormones (which contain very potent progestins) influence fast sleep spindle density and thus sleep‐dependent memory consolidation.

### 4.6 Limitations

First, Genzel and colleagues (2014) found that women using OCs show a significant increase in declarative memory consolidation independent of pill week (pill‐active week or pill‐free week) or nap/wake condition. It seems that in women using OCs, offline memory consolidation increases *per se* irrespective of potential beneficial effects of a nap. In our study we did not have a wake control group with a time period of about 8 h wakefulness between the two retrieval tasks, which would have been required to exclude a general offline effect on declarative memory consolidation (Diekelmann et al., 2009; Payne et al., 2012). In contrast to Genzel and colleagues (2014), we here investigated the influence of menstrual cycle phase and the use of OCs on nocturnal sleep and overnight memory consolidation. Second, to control whether the higher fast spindle density in women using OCs is due to an increase in synthetic progestin levels, also the synthetic progestin level has to be measured in future studies. Although we only included women using OCs containing either ethinylestradiol and levonorgestrel or ethinylestradiol and dienogest, the exact dose of synthetic hormones varies depending on the formulation of the OCs (Lovett et al., 2017). Thus, there is a variability in synthetic hormone exposure (Lovett et al., 2017). Third, to test whether there are also differences between active (intake of synthetic progestins) and inactive pill weeks (no synthetic progestin intake), a group of women using OCs in their inactive pill week should be added as a control (Baker et al., 2001; Genzel et al., 2014). Fourth, the synthetic progestins can bind to androgen receptors, which could contribute to masculinizing (androgenic) effects. Likewise, during the metabolism of the synthetic progestin, it is degraded to testosterone‐like products, which also can have androgenic functions (Pletzer & Kerschbaum, 2014; Pletzer, et al., 2014a). Therefore, to control for androgenic functions of the synthetic progestins, a male‐control group could be added in an upcoming investigation to compare declarative memory performance, consolidation and sleep spindle density between males and females using OCs. Fifth, to determine the cycle phases of our participants, we used self‐reported menstrual cycle questionnaires and did not confirm the menstrual cycle duration by controlling for the onset of next menstruation. Sixth, we used a between‐subjects design instead of a longitudinal within‐subject study design, which would better show whether the menstrual cycle‐dependent fluctuation of progesterone levels effects the variation of spindle density over the menstrual cycle within an individual subject. Seventh, the female sex hormone estradiol also fluctuates across the menstrual cycle and has been positively associated with verbal fluency (Maki et al., 2002), increased offline change in declarative learning and increased spindle density (Genzel et al., 2012) and therefore should be considered in further studies.

## 5 CONCLUSION

We found that naturally cycling women during the luteal phase (characterized by a high endogenous progesterone level) and women using OCs (characterized by a low endogenous progesterone level but with very potent progestins) showed an increased fast spindle density during NREM2 sleep compared to naturally cycling women during the follicular phase (characterized by a low endogenous progesterone level). Along with this, women in the luteal phase as well as women using OCs descriptively showed a highly significant change in memory performance overnight, whereas naturally cycling women during the follicular phase did not. Further, in naturally cycling women during the luteal phase, a high progesterone level was by trend associated with higher fast sleep spindle density. These findings suggest that menstrual cycle‐dependent oscillations in the sex hormone progesterone and application of synthetic sex hormones (containing very potent progestins) affect sleep spindle density and therefore sleep‐dependent memory consolidation. We suggest further research to take the menstrual cycle phase and especially the use of OCs into account when investigating sleep spindles and cognitive performance.

## CONFLICT OF INTEREST

This was not an industry‐supported study. None of the authors has any financial conflict of interest. Georg Gruber is an employee and shareholder of The Siesta Group.

## AUTHOR CONTRIBUTIONS

C.P.P. analysed the data, performed statistical analyses, interpreted the results and drafted the manuscript. H.E.V.W. collected data, analysed the data, performed the statistical analysis, interpreted the results and drafted the manuscript. H.K. designed the hormonal part of the study and interpreted the results. B.A.P. designed the oral contraceptive part of the study design and interpreted the results. G.G. analysed the sleep data. K.O. analysed the hormonal data. M.D. interpreted the results and drafted the manuscript. M.A.H. analysed the sleep data. K.H. designed the study, interpreted the results and drafted the manuscript.

## Supporting information

Supplementary Material

## Data Availability

Data available on request
