# Supplementary material for: Impact of menstrual cycle phase and oral contraceptives on sleep and overnight memory consolidation
Source: J Sleep Res. 2020 Dec 21;30(4):e13239. doi: 10.1111/jsr.13239 (PMC8365641; doi:10.1111/jsr.13239)
Supplement: Supplementary file 1 — Supplementary Material [file JSR-30-e13239-s001.docx]

**Supplementary Material**

**Declarative memory performance**

No group differences were found for traits such as learning ability (VLMT) (R7: (H(2)= 2.398, p= 0.302); ∑R1-5: (F(2,59)= 1.063, p= 0.352, *η_p_^2^=* 0.035), intelligence (IQ) (APM) (F(2,59)= 0.746, p= 0.479, *η_p_^2^=*0.025) or age (H(2)= 1.429, p=0.490).

**Table S1**. Correlations between progesterone level and memory performance and memory consolidation.

|  |  | **progesterone level**  **FOL (n=16)** | **progesterone level**  **LUT (n=27)** | **progesterone level**  **OC (n=19)** |
| --- | --- | --- | --- | --- |
| RET1 | r | -0.181 | -0.171 | -0.102 |
|  | p | 0.502 | 0.394 | 0.678 |
| RET2 | r | -0.234 | -0.169 | -0.042 |
|  | p | 0.382 | 0.398 | 0.863 |
| ΔRET2-RET1 | r | -0.230 | 0.015 | 0.454 |
|  | p | 0.392 | 0.942 | **0.051** |

Abbreviations: FOL: follicular phase; LUT: luteal phase; OC: oral contraceptive use; Slow: sleep spindle density (N/min) for slow spindles (11-13Hz); Fast: sleep spindle density (N/min) for fast spindles (13-15 Hz); RET1=pre sleep recall performance, RET2= post sleep recall performance, ΔRET2-RET1= memory consolidation.

**Table S2**. Correlations between spindle density and memory performance, memory consolidation and trait scores of intelligence (IQ) and learning ability (VLMT).

| **Spindle density** |  | **FOL (n=16)** | | **LUT (n=27)** | | **OC (n=19)** | |
| --- | --- | --- | --- | --- | --- | --- | --- |
|  |  | Fast frontal | Fast central | Fast frontal | Fast central | Fast frontal | Fast central |
| RET1 | r | -0.246 | -0.100 | 0.039 | 0.276 | 0.027 | 0.011 |
|  | p | 0.358 | 0.712 | 0.847 | 0.164 | 0.914 | 0.965 |
| RET2 | r | -0.189 | -0.064 | -0.01 | 0.230 | 0.023 | -0.01 |
|  | p | 0.483 | 0.815 | 0.961 | 0.248 | 0.926 | 0.966 |
| ΔRET2-RET1 | r | 0.235 | 0.152 | -0.302 | -0.288 | -0.027 | -0.164 |
|  | p | 0.382 | 0.574 | 0.125 | 0.145 | 0.911 | 0.503 |
| IQ | r | -0.265 | -0.074 | 0.074 | 0.304 | 0.158 | 0.055 |
|  | p | 0.322 | 0.786 | 0.714 | 0.123 | 0.520 | 0.822 |
| ∑R1-5 | r | -0.203 | 0.065 | 0.268 | 0.270 | -0.007 | 0.075 |
|  | p | 0.451 | 0.811 | 0.177 | 0.173 | 0.977 | 0.761 |
| R7 (r_s_) | r | -0.165 | 0.057 | 0.373 | 0.126 | 0.038 | 0.116 |
|  | p | 0.542 | 0.833 | **0.055** | 0.532 | 0.877 | 0.636 |

Abbreviations: FOL: follicular phase; LUT: luteal phase; OC: oral contraceptive use; Fast: sleep spindle density (N/min) for fast spindles (13-15 Hz); RET1=pre sleep recall performance; RET2= post sleep recall performance; ΔRET2-RET1= memory consolidation; VLMT: ∑R1-5= total learning performance, R7= free recall performance after a 30 minutes time delay. Spearman rho coefficients (r_s_) are reported for correlations with non-normally distributed data.

**Table S3 Post hoc power analyses:**

Dependent sample t-tests, calculated for descriptive statistic, revealed significant higher post sleep memory performance (RET2) compared to pre sleep memory performance (RET1) in women during the luteal phase (t(26)= -3.770, p<0.003, ηp2=0.353), women using OCs (t(18)= -4.008, p= 0.003, ηp2=0.472) but not for women during the follicular phase (FOL: (t(15)= -1.634, p= 0.369, ηp2= 0.151).

To evaluate why the difference between pre- and post-sleep performance was not significant in women during the follicular phase and whether this could be caused by lower statistical power a post hoc power analysis was calculated to compute the achieved power using G*power (Faul et al., 2007). According to the post hoc power analyses each of these dependent t-tests achieved 100% power (cf. Table_S3).

**Table S3.** Post hoc power analyses for naturally cycling women during the follicular phase (FOL) and luteal phase (LUT) and women using OCs (OC).

| **t tests -** Means: Difference between two dependent means (matched pairs)  **Analysis:** Post hoc: Compute achieved power | | | | |
| --- | --- | --- | --- | --- |
|  |  | **FOL (n=16)** | **LUT (n=27)** | **OC (n=19)** |
| **Input:** | Tail(s) | = Two | = Two | = Two |
|  | Effect size dz | =14.0971586 | =20 | =11.3099916 |
|  | α err prob | =0.05 | =0.05 | =0.05 |
|  | Total sample size | =16 | =27 | =19 |
| **Output:** | Noncentrality parameter δ | =56.3886344 | =103.923 | =49.2991104 |
|  | Critical t | =2.1314495 | =2.0555294 | =2.1009220 |
|  | Df | =15 | =26 | =18 |
|  | Power (1-β err prob) | =1.0000000 | =1.0000000 | =1.0000000 |
